# Interspecific visitation of cattle and badgers to fomites: A transmission risk for bovine tuberculosis?

**DOI:** 10.1002/ece3.5282

**Published:** 2019-07-09

**Authors:** Emma L. Campbell, Andrew W. Byrne, Fraser D. Menzies, Kathryn R. McBride, Carl M. McCormick, Michael Scantlebury, Neil Reid

**Affiliations:** ^1^ School of Biological Sciences Queen's University Belfast Belfast UK; ^2^ Veterinary Sciences Division Agri‐Food and Biosciences Institute (AFBI) Belfast UK; ^3^ Veterinary Epidemiology Unit, Department of Agriculture Environment and Rural Affairs (DAERA) Belfast UK; ^4^ Institute for Global Food Security (IGFS) Queen's University Belfast Belfast UK; ^5^ Present address: Department of Agriculture Food and the Marine (DAFM), Agriculture House Dublin 2 Ireland

**Keywords:** camera trapping, disease ecology, *Meles meles*, *Mycobacterium bovis*, surveillance, wildlife–livestock interface

## Abstract

In Great Britain and Ireland, badgers (*Meles meles*) are a wildlife reservoir of *Mycobacterium bovis* and implicated in bovine tuberculosis transmission to domestic cattle. The route of disease transmission is unknown with direct, so‐called “nose‐to‐nose,” contact between hosts being extremely rare. Camera traps were deployed for 64,464 hr on 34 farms to quantify cattle and badger visitation rates in space and time at six farm locations. Badger presence never coincided with cattle presence at the same time, with badger and cattle detection at the same location but at different times being negatively correlated. Badgers were never recorded within farmyards during the present study. Badgers utilized cattle water troughs in fields, but detections were infrequent (equivalent to one badger observed drinking every 87 days). Cattle presence at badger‐associated locations, for example, setts and latrines, were three times more frequent than badger presence at cattle‐associated locations, for example, water troughs. Preventing cattle access to badger setts and latrines and restricting badger access to cattle water troughs may potentially reduce interspecific bTB transmission through reduced indirect contact.

## 1 INTRODUCTION

Bovine tuberculosis (bTB) caused mainly by the infectious agent, *Mycobacterium bovis*, has been a disease of high economic importance in veterinary and human medicine for decades (Gordejo & Vermeersch, 2006). Management of the disease in domestic cattle costs the United Kingdom (UK) over £130 million per year covering surveillance testing, slaughter of infected animals, and compensation to the affected farmers (DAERA, 2017; DEFRA, 2016). A bTB herd breakdown increases direct costs to the farmer through supplementary feeding, inability to trade (i.e., a ban on animal movements), the expense of preparing for subsequent bTB testing, as well as the implications to farmers mental health due to herd disease status uncertainty (Robinson, 2017). Consequently, bTB has been the focus of ongoing national, and various regional, eradication programs in both the UK and Ireland for over 50 years. Despite these programs, bTB remains a significant burden due to its complex epidemiology (Allen, Skuce, & Byrne, 2018).

Wildlife reservoir hosts of *M. bovis* circulate infection within populations and can pose a risk to domestic livestock hosts (Fitzgerald & Kaneene, 2013). Prominent wildlife reservoir hosts include European badgers (*Meles meles*) in the UK and Ireland (Krebs et al., 1997; Murphy, Gormley, Costello, O'Meara, & Corner, 2010; Woodroffe et al., 2005), brush‐tailed opossums (*Trichosurus vulpecula)* in New Zealand (Morris & Pfeiffer, 1995) and white‐tailed deer (*Odocoileus virginianus)* in Michigan, USA (O'Brien et al., 2002). Understanding reservoir infection dynamics is essential if the disease is to be controlled, indeed Haydon, Cleaveland, Taylor, and Laurenson (2002) concluded disease‐control measures should be directed at all reservoir populations if full eradication is to be achieved. Understanding how badgers and cattle may come into contact with each other will help influence these disease‐control measures.

A number of lines of evidence implicate badgers in the epidemiology of bTB in cattle populations. Culling trials, for example, demonstrated a reduced risk of cattle herd breakdown where badgers had been culled relative to control populations (Donnelly et al., 2006; Griffin et al., 2005). Badgers and cattle carry the same molecular type of *M. bovis* locally; however, the pathways of transmission are not clear (Griffin, Martin, Thorburn, Eves, & Hammond, 1996; More & Good, 2006; Woodroffe et al., 2005). Despite this, interspecific transfer is suggested by high‐resolution genomic data on localized wildlife‐cattle bTB strains (Biek et al., 2012). Cattle bTB outbreaks are more likely when badgers are present within close proximity (for example, <1.5 km) of a farm (Byrne et al., 2015; Denny & Wilesmith, 1999; Martin et al., 1997; Vial, Johnston, & Donnelly, 2011), and large‐scale studies have found associations in badger social group abundance and elevated herd risk (Bessell, Orton, White, Hutchings, & Kao, 2012; Byrne, White, McGrath, O'Keeffe, & Martin, 2014; Wright et al., 2015). The UK and Irish Governments have responded by advocating culling and/or vaccinating badger populations to reduce bTB risk (DEFRA, 2016; More & Good, 2006). Interestingly, Mathews, Lovett, Rushton, and Macdonald (2006) found reduced bTB risk when farms were managed to maintain wildlife habitat, which may offer badgers a refuge reducing their potential contact with livestock.

*Mycobacterium bovis* is thought to be transmitted predominately through aerosols, and transfer is thought to occur between cattle via so‐called “nose‐to‐nose” contact. Transmission is also possible through bite wounds and contact with urine and feces of infected animals (Buddle, Aldwell, Pfeffer, Lisle, & Corner, 1994; Menzies & Neill, 2000; Neill, Hanna, O'Brien, & McCracken, 1988). Direct contact between badgers and cattle via nose‐to‐nose contact can occur (Tolhurst, Delahay, Walker, Ward, & Roper, 2009), but studies suggest this is extremely rare with some suggesting that badgers actively avoid pastures when cattle are present, with close physical proximity almost never recorded (Benham & Broom, 1989; Böhm, Hutchings, & White, 2009; Mullen et al., 2013; O'Mahony, 2014a; Woodroffe et al., 2009). Thus, direct contact between live badgers and cattle seems to be an unlikely route to maintain disease through interspecific transmission.

Indirect contact, in which badgers and cattle use the same space but at different times, may result in the potential transfer of infectious material via fomites, such as contaminated soils, water, or feedstuffs. Badgers use latrines for defecation and urination which may act as a focal point for *M. bovis* accumulation (Hutchings & Harris, 1997). *M. bovis* has been found in the soil surrounding badger setts and latrines (Courtenay et al., 2006; Sweeney et al., 2007) where concentrations are higher than in adjacent pasture (Young, Gormley, Wellington, Elizabeth, & Wellington, 2005). Badgers can excrete *M. bovis* through sputum, urine, feces, discharging infected wounds, and through nasal secretions (Clifton‐Hadley, Wilesmith, & Stuart, 1993; Corner, Murphy, & Gormley, 2011). Badger urine can contain more bacilli (217 × 10^3^ cfu/ml) than bronchial pus (73 × 10^3^ cfu/ml) or feces (68/g); therefore, contamination of pasture and soils with infected urine may pose the greatest risk of indirect infection (Courtenay et al., 2006; Gallagher & Horwill, 1977).

Over half (56%) of mud samples near water sources in Ireland tested positive for *M. bovis* by real‐time PCR, with other investigations suggesting bacilli can remain viable in damp soil for up to 15 months (Fine, Bolin, Gardiner, & Kaneene, 2011; Ghodbane, Medie, Lepidi, Nappez, & Drancourt, 2014; Young et al., 2005). Therefore, fomites may potentially transfer infection to new animals long after infected animals have left the vicinity. Badgers have been recorded using farm buildings in Great Britain where they may actively forage (Garnett, Delahay, & Roper, 2002; Tolhurst et al., 2009). Judge, McDonald, Walker, and Delahay (2011) deployed cameras at 32 farms for 365 days, and badgers were recorded at 19 (59%). Badger feces and urine have been reported in feed stores or close to water troughs in the field (Garnett et al., 2002; Garnett, Roper, & Delahay, 2003; Tolhurst et al., 2009).

Given the accumulating evidence against direct badger‐cattle nose‐to‐nose transmission, it may be hypothesized that indirect transmission via contaminated fomites is likely the most epidemiologically significant infection route. Thus, this study aimed to understand potential transmission risk by quantifying (a) badger visitation to cattle sites and (b) cattle visitation to badger sites using camera traps at focal locations both in the farmyard and at pasture. The temporal lag between cattle and badgers using the same space at different times was also quantified.

## 2 METHODS

### 2.1 Study site

The study was undertaken in Co. Down, Northern Ireland (NI), in an area which has high cattle density, high badger habitat suitability, high badger sett density (DAERA, 2017; Reid, Etherington, Wilson, McDonald, & Montgomery, 2008) and within a so‐called “bTB hotspot” with records of herd breakdowns for many years (DAERA, 2017; Wright et al., 2015). The drumlin (rolling hills) landscape was predominately improved grassland grazed by cattle or used for silage production with some sheep grazing and a small proportion of interspersed arable land. A total of 34 farms participated in the study with 14 (41%) dairy and 20 (59%) beef production systems. The farms were all < 10 km from one another to reduce geographical sources of variation. Median farm size was 41.8 ha, and median herd size was 83.3 cattle. The landscape and farming systems were typical pastoral farming in Northern Ireland where the vast majority of badger setts are located within farmland hedgerows (Byrne, Sleeman, O'Keeffe, & Davenport, 2012; Menzies, Abernethy, Stringer, & Jordan, 2011).

### 2.2 Quantification of visitations rates

Camera trapping is a useful method in quantifying species‐specific space‐use when conducted at focal sites of interest (Caravaggi et al., 2018), in this case, likely bTB fomites. Operating 24 hr a day for 7 days, remote cameras provide continuous survey effort at each location and generate large volumes of footage to process (Swann, Kawanishi, & Palmer, 2011).

Remote camera traps (Bushnell Trophy Cam HD cameras; model 119,477) were placed at approximately 75 cm above ground level and used to quantify animal visitation rates to different sites containing putative bTB fomites (substrates potentially contaminated by *M. bovis*).

The aim was to deploy a camera trap at six locations on each farm. To quantify each species presence at badger locations, visitation rates were enumerated at three so‐called “badger locations”: a run, a latrine, and a sett (which were perceived to represent an ordinal scale of risk from low to high). At setts, cameras were preferentially placed at an active main sett. Before deploying cameras, each farm was surveyed to locate active runs, latrines, and setts (following Reid, Etherington, Wilson, Montgomery, & McDonald, 2011).

To quantify each species presence at cattle locations, visitation rates were enumerated at three so‐called “cattle locations”: cattle shed entrance, feed store entrance, and a water trough (which were perceived to represent an ordinal scale of risk from low to high). Farm building entrances were chosen at those buildings housing cattle during winter. Feed locations were either at feed bins or dispensed whole crop silage. Water troughs were not located within farmyards but usually at a pasture field boundary. Typically, a water trough was a low‐level concrete basin kept filled with water via a ballcock system and readily accessible by wildlife as well as cattle.

Each camera recorded video footage in 720‐pixel resolution for a duration of 20 s and had an interval of 1 min between recordings. Cameras recorded 24 hr a day for 7 consecutive days and recorded from 6 p.m. on the first day to 8 a.m. on the last day. A trial was performed on 4 of the farms at the 6 proposed locations to check that no avoidance or attraction behavior was exhibited. There was no discernible difference in animal visitation rates or behaviors during the 7 days of deployment. Subsequently, each camera was left in situ for 158 hr per survey period. Data were collected during two periods of the year; winter–spring (February to March 2016) when cattle were housed and summer–autumn (July to September 2016) when cattle were grazing in fields. Taking both survey periods together, cameras were deployed on each of the 34 farms for 1,896 hr (totaling 64,464 hr of survey effort).

All footage was viewed and the presence and type of any mammal or bird recorded. To avoid double counting of multiple videos of the same detection event, an interval of >10 min between videos of the same species was used to define unique events. A 10‐min interval between videos was chosen after initially reviewing footage. Where multiple recordings occurred after an initial triggering event, the number of subsequent (repeat) recordings appeared to decrease after ~10 min. Duplicate records were deleted prior to analysis.

### 2.3 Environmental and BTB data

The Met Office (www.metoffice.gov.uk) provided daily weather data (minimum temperature, total precipitation, and wind speed) from the Katesbridge automatic weather station (latitude and longitude: 54°17′49.2″, −6°6′36.0″) located 3.5 km from the centroid of the study site. The Department of Agriculture, Environment and Rural Affairs (DAERA) provided for the number of bTB cattle herd breakdowns on each farm for the 1‐, 3‐, and 5‐year periods prior to the camera trap survey.

### 2.4 Statistical analyses

Basic descriptive statistics were used to report camera trapping results. Each record in the dataset represented 1 unique observation recorded during the week of camera deployment. Badger visitation rates, that is, the frequency of badgers detected per week were analyzed using a generalized linear mixed model (GLMM) in the R package *glmmADMB* 0.8.0 (Bolker et al., 2009). Explanatory variables were checked for multicollinearity using Spearman's rank correlations. Badger visitation frequency was used as the dependent variable and was assumed to have a negative binomial distribution fitted using a log link function. A negative binomial distribution was chosen as the variance within the outcome was greater than the mean, making it a better option than the more traditional Poisson distribution. Farm was fitted as a random factor to account for multiple observations per farm. The global model fitted season, location, production type, and the presence or absence within the week of camera deployment of; cats, cattle, dogs, farmers, squirrels, mice, foxes, rabbits, and sheep, as fixed factors and herd size, the frequency of birds, meteorological conditions including minimum temperature, rainfall and speed wind, and the number of bTB reactors confirmed in the 1‐, 3‐, and 5‐year periods prior to the study as covariates. A forward and backward elimination of variables was used to obtain the best approximating single model using the Akaike information criterion (AIC) as the selection metric (Dohoo, Martin, & Stryhn, 2003). Interaction terms were fitted initially but removed as their presence increased AIC values. A Wilcoxon sign rank test was used to compare the frequency of both badger and cattle detections at badger runs. A negative binomial GLMM was also performed on the frequency of cattle detections as the dependent variable. Again, farm was fitted as a random factor. The global model in this case contained season, location, production type, herd size, and farmer presence. All statistics were performed using R v3.4.2 (R Development Core Team & R, 2017).

## 3 RESULTS

Due to access being denied at some sites and camera failures, we had a final sample of 28 latrines, 34 runs, and 32 setts (i.e., 94 badger locations) and 29 farm building entrances, 24 feed stores, and 31 water troughs (i.e., 84 cattle locations). Camera traps made 68,320 motion detections (totaling 380 hr of footage), of these 27,732 detections (totaling 154 hr of footage) contained animals, farmers, or farm machinery (Table 1).

**Table 1:** Detection frequency of each species on farms in Co. Down, Northern Ireland, during 2016

Species	Captures		
	Total	Unique	%
Bird	8,856	4,858	35.7
Farmer	5,100	2,376	17.5
Cat	1,849	1,327	9.8
Rabbit	1,741	1,025	7.5
Cattle	4,566	974	7.2
Sheep	2,485	904	6.6
Badger	1,213	889	6.5
Dog	842	396	2.9
Fox	354	341	2.5
Mouse	364	261	1.9
Rat	302	192	1.4
Squirrel	45	43	0.3
Pig	5	4	0.03
Donkey	4	3	0.02
Hare	4	3	0.02
Hedgehog	2	2	0.01
Total	27,732	13,598	100.00

A total of 1,213 badger detections were recorded, of which 889 were unique detections (more than 10 min between videos of the same species). A total of 4,566 cattle detections were recorded, of which 974 were unique detections.

**Figure 1:**
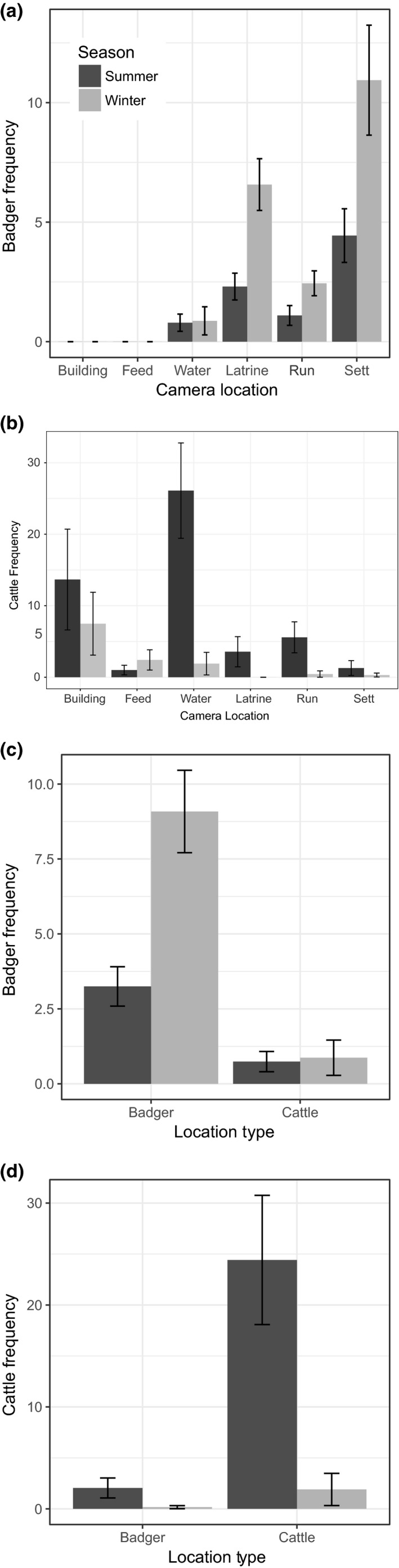
(a) Badger and (b) cattle weekly visitation rates ± 1 standard error at each of six locations per farm. (c) Badger and (d) cattle seasonal visitation rates ± 1 standard error at badger locations (latrines and setts only) and cattle locations (water troughs only)

Badgers were never detected within a farmyard whether at farm building entrances or feed stores regardless of season (Figure 1a). However, there were 76 unique detections of foxes entering farmyards on 13 of the farms. Farm cats were notably common with 1,327 unique detections. In farmyards (buildings and feed stores), there was a median of 3 cat detections per week (range 0–283 cat detections per week).

On each farm, there was a median of 8.5 badger detections per week. The total number of badger detections at each farm over both seasons ranged from 1 to 69 unique detections (Table 2). Badger detections varied between camera locations (Table 3a), with visitation rates being highest at setts followed by latrines, then runs, and finally water troughs (Figure 1a).

**Table 2:** Frequency of badger visits (number of unique detections in summer and winter totaling 14 nights) per study farm during 2016

Badger visits per farm	Number of farms	%
0–10	10	29
11–20	7	21
21–30	5	15
31–40	3	9
41–50	3	9
51–60	4	12
61–70	2	6

**Table 3:** Results of the best fitting negative binomial generalized linear mixed model (GLMM) with (a) badger and (b) cattle detections as the dependent variable, farm fitted as a random factor

Variable	χ^2^	*df*	*β* ± *SE*	95% CI (Lower, Upper)	*p*
(a) Badger detections					
Location	120.642	3	Factorial		<0.001
Latrine vs Sett			−0.427 ± 0.267	−0.951, 0.096	0.110
Run vs Sett			−1.594 ± 0.271	−2.125, −1.062	<0.001
Water vs Sett			−2.302 ± 0.305	−2.901, −1.703	<0.001
Season	11.758	1	Factorial		<0.001
Summer vs Winter			−0.754 ± 0.220	−1.185, −0.323	0.001
Fox presence	7.830	1	0.615 ± 0.220	0.184, 1.045	0.005
Cattle presence	4.416	1	−0.751 ± 0.357	−1.451, −0.051	0.036
Herd size	3.567	1	0.002 ± 0.001	−0.00006, 0.003	0.059
Sheep presence	2.960	1	0.550 ± 0.320	−0.077, 1.176	0.085
Rabbit presence	2.572	1	0.365 ± 0.228	−0.081. 0.811	0.109
(b) Cattle detections					
Location	28.445	5	Factorial		<0.001
Building vs Water			−0.715 ± 1.113	−2.898, 1.467	0.521
Feed vs Water			−2.359 ± 1.138	−4.590, −0.128	0.038
Latrine vs Water			−2.489 ± 0.830	−4.115, −0.864	0.003
Run vs Water			−1.573 ± 0.757	−3.057, −0.088	0.038
Sett vs Water			−2.190 ± 0.901	−3.955, −0.424	0.015
Season	18.215	1	Factorial		<0.001
Summer vs Winter			2.882 ± 0.675	1.559, 4.206	<0.001
Farmer present	8.167	1	2.582 ± 0.904	0.811, 4.353	0.004

Badger detections also varied between seasons (Table 3a) with 2.6 times more detections during winter, when cattle were housed, than during summer, when cattle were grazing. This was the case at runs, latrines, and setts but apparently not at water troughs where badger visitation rates appeared similar between summer and winter (Figures 1a,c, & 2a); there was, however, no statistical difference when this was applied in the model.

**Figure 2:**
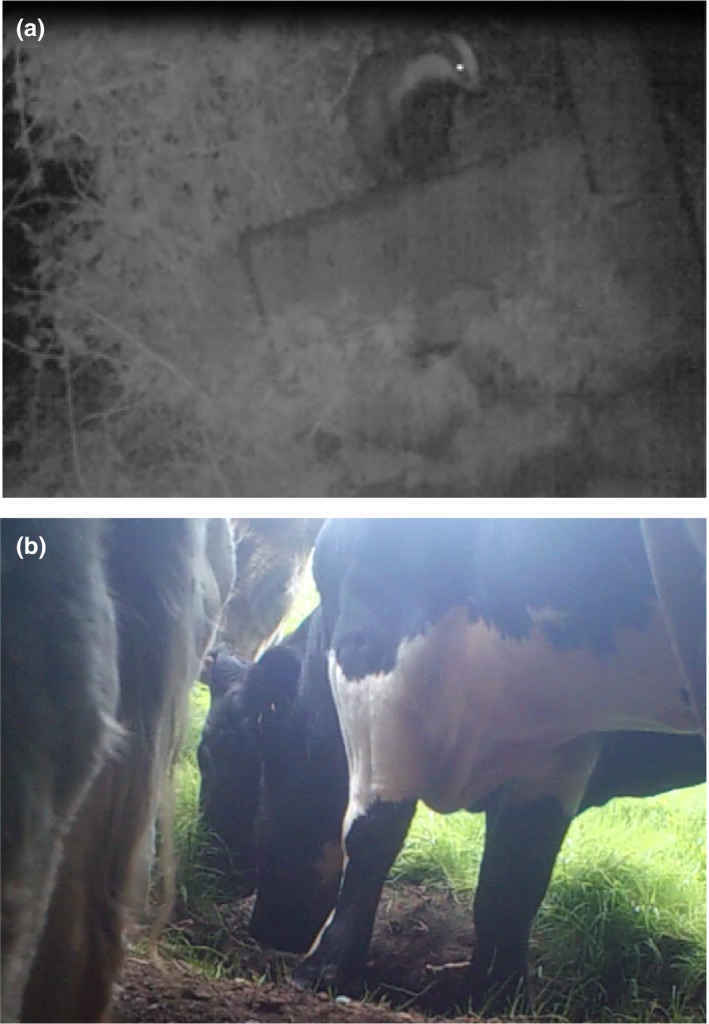
Camera trap still images of (a) badger at a cattle water trough and (b) cattle at a badger sett entrance

**Figure 3:**
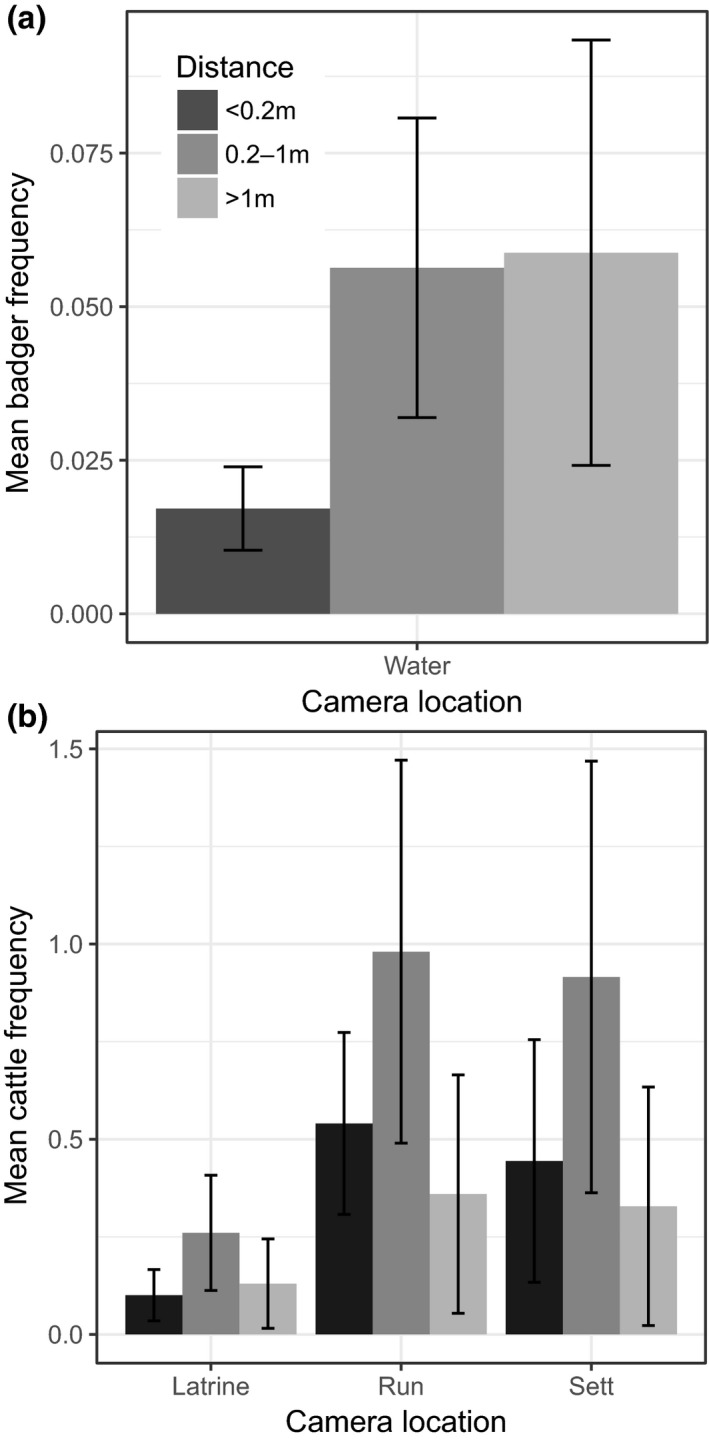
Mean daily estimated proximity ± 1 standard error in both seasonal surveys combined of (a) badgers at cattle locations (at water troughs only) and (b) cattle at badger locations (latrines, runs, and setts)

Badgers visited the vicinity of water troughs on average 0.8 (95%CI 0.1–1.5) times per week throughout both seasons. There were 50 badger detections nearby water troughs but animals were in direct contact with the trough on only 10 occasions (Figure 3a), and only observed drinking directly on 5 occasions, that is, 1 badger was detected drinking at a water through every *ca*. 87 camera days (Table 4a).

**Table 4:** (a) Badger behavior when in proximity of cattle locations and (b) Cattle behavior when in proximity of badger locations

Directionality of indirect contact		Frequency of detection			Total
		Distance			
Cattle location	Badger behavior	<0.2 m	0.2–1.0 m	>1.0 m	
(a) Badger activity					
Water	Drinking	5	0	0	5
	Foraging	0	4	16	20
	Urinating/defecating	0	1	0	1
	Walking	5	12	7	24
	Total	10	17	23	50

Badger location	Cattle behavior				
(b) Cattle activity					
Latrine	Foraging	6	7	6	19
	Grazing	11	9	26	46
	Walking	0	13	4	17
Run	Foraging	47	72	20	139
	Grazing	0	12	5	17
	Walking	18	9	5	32
Sett	Foraging	2	9	2	13
	Grazing	0	0	1	1
	Sniffing	6	0	0	6
	Walking	0	30	1	31
	Total	90	161	70	321

Note

Foraging = animals head down and looking for food; grazing = actively consuming food.

The best fitting model suggested that badger detection was positively associated with herd size and fox, sheep, and rabbit presence and negatively associated with cattle presence (Table 3a). Badger detection was not associated with farm production system (i.e., dairy or beef), farmer activity, the frequency of other species detected, weather parameters (minimum temperature, precipitation, or wind speed), or the bTB history of the farm during the previous 1‐, 3‐, or 5‐year period.

Cattle were detected more frequently during summer than winter (Table 3b), most notably at water troughs (Figure 1b). Cattle were observed more frequently at feed stores during winter than summer. During the summer months, there was no difference in the number of detections of cattle and badgers at badger runs (Wilcoxon rank W = 590, *p* = 0.863, *n* = 64).

Cattle visits to badger locations were more frequent during summer when cattle where grazing than during winter when housed (Figure 1d). During summer, there was the equivalent of 1 individual cattle visit to a badger latrine or sett 0.3 times a day (*ca*. 1 visit every three days) while badgers visiting cattle locations (water troughs only) 0.1 times daily (*ca*. 1 visit every 10 days). Therefore, cattle visits to badger locations were *ca*. 3.3 times more frequent than badgers visiting cattle locations.

Cattle were most frequently observed < 1 m from badger locations (latrines and setts) with 65 close contacts with runs, 17 with latrines, and 8 with a sett (Figure 3b). Cattle typically grazed when near runs and latrines, with 6 cows observed sniffing sett entrances (Figure 2b; Table 4).

Badgers and cattle were never recorded at the same location at the same time, that is, were never observed in the same 20‐s video clip. Nevertheless, they did occupy the same locations at different times. The mean interval between a cattle detection followed by a badger detection was 9.2 hr (ranging from 1.5–23.9 hr). The mean interval between a badger detection followed by a cattle detection was 17.2 hr (ranging from 0.7 to 57.1 hr). This pattern generally held regardless of whether the location was a run, latrine, or water trough. However, at setts the interval between visits for both cattle–badger and badger–cattle were both approximately 10 hr (Table 5). At water troughs, the mean interval between a cattle detection followed by a badger detection was 20.5 hr (ranging from 7.4 to 33.6 hr) while the interval between a badger detection followed by a cattle detection was 8.3 hr (ranging from 1.5 to 15.0 hr).

**Table 5:** Time interval between cattle‐badger and badger‐cattle visitation at different farm locations

Location	Interaction	Frequency	Mean interval (hr) ± *SD*	Interval range (hr)
Latrine	Badger–cattle	4	29.8 ± 18.5	16.1–57.0
	Cattle–badger	2	14.4 ± 13.3	5.0–23.9
Run	Badger–cattle	5	18.5 ± 17.5	3.2–47.8
	Cattle–badger	3	4.1 ± 1.4	2.9–5.7
Sett	Badger–cattle	10	10.9 ± 11.6	0.7–40.2
	Cattle–badger	7	10.2 ± 4.3	4.3–17.0
Water	Badger–cattle	2	20.5 ± 1.8	7.4–33.6
	Cattle–badger	2	8.3 ± 9.5	1.5–15.0

## 4 DISCUSSION

In the current study, cattle and badgers certainly visited fomites associated with the other species. Some previous studies with more focus on visitation to farmyards have asserted that badgers forage at cattle feed locations (Payne, Chappa, Hars, Dufour, & Gilot‐Fromont, 2015; Tolhurst et al., 2009; Woodroffe et al., 2017), that badgers visit farm yards frequently (Garnett et al., 2002; Judge et al., 2011), and that direct contact between the species may be highest at farm buildings (Tolhurst et al., 2009). However, badgers never entered farmyards in this current study, a finding also reported by other recent studies from Northern Ireland (O'Mahony, 2015), the Republic of Ireland (Mullen et al., 2015; Sleeman, Davenport, & Fitzgerald, 2008), and in Great Britain (Woodroffe et al., 2017).

Farm biosecurity was frequently breached by wildlife other than badgers, such as foxes, suggesting that if badgers had been present, they would have been detected. Further study may be warranted, with longer camera deployments throughout the year to confirm whether the absence of badgers is consistent across all seasons. For example, a previous study with camera deployment over a period of 1 year found badgers had the least contribution to intrusion rates relative to other wild animals (O'Mahony, 2015). This appears to suggest that there is substantial variation in farmyard visitation rates across study populations. While this could be partly attributed to differences in survey effort used to assess visitation rates, there may also be ecological factors impacting visitation rates including badger density (Mullen et al., 2015; O'Mahony, 2014a; Woodroffe et al., 2017) which potentially effects badger foraging choices and behavior. Higher badger density appears to correlate with increased farmyard visitation rates (Mullen et al., 2015; O'Mahony, 2014a; Woodroffe et al., 2017).

At the locations of the camera traps, badgers and cattle were never recorded at the same time and location. In studies using proximity loggers, badger and cattle close contact was found to be very rare (Drewe, O'connor, Weber, McDONALD, & Delahay, 2013; O'Mahony, 2014b; Woodroffe et al., 2016). Therefore, it is likely that camera trapping at point locations may be limited in its ability to capture such rare events.

Badger activity was positively associated with fox and rabbit presence probably due to their similar, generalist, habitat requirements, that is, an association with hedgerow boundaries for the construction of setts, burrows, and warrens (Macdonald et al., 2004), however badgers and foxes exhibit competition for resources (Trewby et al., 2008).

All farms had at least one badger detection suggesting that badgers may pose a biosecurity risk at all farms surveyed. Nevertheless, we found a negative relationship between badger activity and cattle presence consistent with badgers avoiding pasture with grazing cattle (Benham & Broom, 1989; Mullen et al., 2013; Woodroffe et al., 2016). In contrast, badgers did not avoid and exhibited a positive association with grazing sheep. Badgers were detected more frequently during winter when cattle were housed. In winter, there is low natural food availability and longer hours of darkness facilitating greater badger activity.

Badgers were detected in the vicinity of cattle water troughs during winter and summer with equal frequency but were observed drinking rarely (equivalent to 1 detection per water trough every 87 days). Previously, badgers have been observed utilizing cattle water troughs by standing on their hind limbs (O'Mahony, 2014b) but in the present study animals were observed standing on top of water trough reservoirs or by leaning in from adjacent hedgerow banks which provided access (Figure 2a). Water troughs were the sole locations where badgers visited cattle locations. This suggests that if badger access to open water sources could be restricted, it would virtually eliminate badgers visiting so‐called cattle locations. Raising water troughs off the ground and placing them away from adjacent access points (e.g., hedgerow banks) would likely reduce badger access, but it may not eliminate it entirely, as badgers are known to climb (Garnett et al., 2003). DEFRA recommend water troughs being at least 90 cm above ground level, and they also recommend ensuring troughs are inaccessible to badgers. It may be worth monitoring natural drinking locations (streams, rivers, and ponds) for cattle visitations, to assess whether restricting cattle access is needed (Barasona et al., 2016).

In this study, there was no relationship between badger activity and bTB status of farms in the previous 1, 3, or 5 years. Suggesting that any risk due to indirect contact between badgers and cattle appears sufficiently low as to have an undetectable effect using the current survey methods. However, such a relationship would be difficult to demonstrate with the 34 farms in the study and the relatively low incidence rates within the study (i.e., low statistical power).

Cattle visited badger locations over three times as frequently as badgers visited cattle locations, with cattle visiting badger latrines or setts on average once every 3 days. These figures are derived from unique detections only, and thus, some cattle may well have spent prolonged periods investigating fomites, but this was not formally captured due to the deletion of duplicate detections within the same 10‐min period after each camera triggering event. A future study could investigate these prolonged visits to fomites and the behavior exhibited by both species while present. While badgers use latrines, they may also deposit urine in their runs while on the move (Hutchings, Service, & Harris, 2002). Cattle–badger indirect contact at runs may, therefore, play a role in disease spread, and cattle are known to graze (or at least not to avoid) pasture contaminated with badger urine (White, Brown, & Harris, 1993). Restricting cattle access to badger runs which may bisect fields used for grazing is likely to be unfeasible.

Cattle were observed investigating badger setts by sniffing entrances and inserting their muzzles into burrows. If these setts were occupied by badgers infected with and excreting *M. bovis*, these cattle may inhale infectious material. Young et al. (2005) found bacilli were present at setts more frequently, and survived longer, than bacilli on pasture on the same farm. Thus, cattle sniffing (and potentially rubbing) behavior in‐and‐around sett entrances may be enough to aerosolize bacilli allowing them to be inhaled.

Farmers can accurately report badger presence and activity levels on their farm when badgers are present, but their observations are vulnerable to type II errors, that is, the reporting of false absences when badgers are in fact present (Menzies et al., 2011; O'Hagan, Matthews, Laird, & McDowell, 2016). If farmers are capable of identifying badger activity, they may also be capable of identifying potential locations where badger–cattle contact could occur and take the necessary action to minimize any risk of indirect bTB transmission by excluding cattle, for example, by fencing off badger setts.

Drewe et al. (2013) recorded 400 visits by badgers and 1,700 visits by cattle to badger latrines. They suggested that these contacts could be an important mechanism in indirect transmission at pasture. The data had network analysis performed by Silk et al. (2018) who found that latrines were not only a potential site for badger‐to‐badger transmission between different social groups but also a point of contact for badger‐to‐cattle transmission. They concluded that not all latrines were equal and that some tended to have more, and stronger connections, to both badgers and cattle. As latrines are frequently close to linear boundaries, maintaining field edge margins of longer sward or fencing off field edge margins may reduce cattle–badger indirect contact further. Cattle are more likely to graze contaminated pasture where sward length is short (Hutchings & Harris, 1997). This practice pushes cattle to graze field boundaries where they are more likely to encounter a latrine or sett. Restricting cattle access to badger setts and latrines may be important with respect to farmers using cattle to “clean up” field margins after silage has been harvested (Ward, Judge, & Delahay, 2010).

Despite the removal of duplicate detections, in almost all cases (excluding water troughs) badgers encountered potential cattle‐deposited fomites approximately twice as quickly (*ca*. 9‐hr interval) as cattle encountered potential badger‐deposited fomites (*ca*. 17‐hr interval). Thus, any infectious agent deposited by cattle may be exposed to the environment for about half as long as any infectious agent deposited by a badger before the next potential host makes contact. These data may not be epidemiologically meaningful if bacilli can survive in the environment for several months (Ghodbane et al., 2014; Young et al., 2005). Nevertheless, cattle‐deposited fomites seem likely to be fresher when encountered by a badger subsequently than badger‐deposited fomites when encountered by cattle subsequently.

Preventing badgers visiting cattle locations by restricting badger access to water troughs by raising them off the ground or installing pasture pumps, and preventing cattle visits to badger locations by fencing off setts and/or field edge margins where latrines are most likely to occur are low‐tech solutions that are within the skills set of every farmer. Regional badger culling programmes are controversial due to political debates over their expense, efficacy, and animal welfare implications (Lederman, 2016) resulting in much public opposition. Equally, badger bTB vaccination trials involve similar expense in terms of badger trapping and vaccine administration with the outcomes (in terms of risk reduction to cattle) yet to be determined. Thus, while both culling and vaccination have, or are being, trialed, we advise consideration of a low‐tech, low‐cost trial to test the efficacy of restricting badger–cattle, and more notably, cattle–badger contact.

This study quantified visitation rates of cattle and badgers to potential *M. bovis* fomites. Cattle did visit badger locations, therefore attempts should be made to minimize cattle contact with badger sites as much as is feasibly possible. Caution should be exercised as a small number of focal locations were surveyed per farm with camera placement critical in terms of quantifying interspecific visitation rates. A large‐scale study over extended timeframes in multiple geographic regions investigating indirect contacts at fomites is needed to assess risk of cattle bTB herd breakdowns, including the sampling of fomites for viable bacilli and through the association of known infection status of hosts in the area (i.e., both badgers and cattle). Moreover, a pathogenesis mechanism for indirect transmission of *M. bovis* has still to be elucidated.

## AUTHOR CONTRIBUTION

EC, NR, FM, AWB, and MS designed the study. EC, KMB, and CMC collected the data; EC analyzed the data and drafted the manuscript; FM provided the herd TB data. NR and AB helped with statistical analysis. NR helped with redrafting the manuscript. All authors contributed to the final version and approved the manuscript.

## Data Availability

The data are available at https://doi.org/10.5061/dryad.mj58cj6.
